# Triage of high-risk HPV-positive women in population-based screening by miRNA expression analysis in cervical scrapes; a feasibility study

**DOI:** 10.1186/s13148-018-0509-9

**Published:** 2018-06-07

**Authors:** Iris Babion, Barbara C. Snoek, Putri W. Novianti, Annelieke Jaspers, Nienke van Trommel, Daniëlle A. M. Heideman, Chris J. L. M. Meijer, Peter J. F. Snijders, Renske D. M. Steenbergen, Saskia M. Wilting

**Affiliations:** 1 Cancer Center Amsterdam, Department of Pathology, VU University Medical Center, Amsterdam, The Netherlands; 2 Department of Epidemiology and Biostatistics, VU University Medical Center, Amsterdam, The Netherlands; 3 Center for Gynaecological Oncology, Antoni van Leeuwenhoek Hospital/Netherlands Cancer Institute, Amsterdam, The Netherlands; 4 Department of Medical Oncology, Erasmus MC Cancer Institute, Erasmus University Medical Center, Rotterdam, The Netherlands

**Keywords:** miRNA, HPV, Cervical cancer, CIN, Scrape, Triage, Screening, qRT-PCR

## Abstract

**Background:**

Primary testing for high-risk HPV (hrHPV) is increasingly implemented in cervical cancer screening programs. Many hrHPV-positive women, however, harbor clinically irrelevant infections, demanding additional disease markers to prevent over-referral and over-treatment. Most promising biomarkers reflect molecular events relevant to the disease process that can be measured objectively in small amounts of clinical material, such as miRNAs. We previously identified eight miRNAs with altered expression in cervical precancer and cancer due to either methylation-mediated silencing or chromosomal alterations. In this study, we evaluated the clinical value of these eight miRNAs on cervical scrapes to triage hrHPV-positive women in cervical screening.

**Results:**

Expression levels of the eight candidate miRNAs in cervical tissue samples (*n* = 58) and hrHPV-positive cervical scrapes from a screening population (*n* = 187) and cancer patients (*n* = 38) were verified by quantitative RT-PCR. In tissue samples, all miRNAs were significantly differentially expressed (*p* < 0.05) between normal, high-grade precancerous lesions (CIN3), and/or cancer. Expression patterns detected in cervical tissue samples were reflected in cervical scrapes, with five miRNAs showing significantly differential expression between controls and women with CIN3 and cancer. Using logistic regression analysis, a miRNA classifier was built for optimal detection of CIN3 in hrHPV-positive cervical scrapes from the screening population and its performance was evaluated using leave-one-out cross-validation. This miRNA classifier consisted of miR-15b-5p and miR-375 and detected a major subset of CIN3 as well as all carcinomas at a specificity of 70%. The CIN3 detection rate was further improved by combining the two miRNAs with HPV16/18 genotyping. Interestingly, both miRNAs affected the viability of cervical cancer cells in vitro.

**Conclusions:**

This study shows that miRNA expression analysis in cervical scrapes is feasible and enables the early detection of cervical cancer, thus underlining the potential of miRNA expression analysis for triage of hrHPV-positive women in cervical cancer screening.

**Electronic supplementary material:**

The online version of this article (10.1186/s13148-018-0509-9) contains supplementary material, which is available to authorized users.

## Background

Cervical cancer screening by cytological examination of cervical scrapes has largely reduced the incidence and mortality rates of cervical cancer in developed countries due to early detection of well-recognizable and treatable precursor lesions (cervical intraepithelial neoplasia (CIN), graded 1–3) [1]. Persistent infection with high-risk types of the human papillomavirus (hrHPV) is a necessary cause of cervical cancer [2–4]. Testing for hrHPV DNA has been demonstrated to have a higher sensitivity for the detection of cervical high-grade CIN and cancer than the cytology-based Pap smear [5, 6]. Consequently, hrHPV testing has recently been implemented as primary screening method in The Netherlands and various other countries. As hrHPV testing also detects women with clinically irrelevant transient infections, additional triage markers are required to identify women with high-grade CIN lesions who are in need of treatment given their risk of developing cancer. Cytology is the currently recommended triage strategy for hrHPV-positive women. An ideal triage test, however, should be objective (non-morphological), available in a high-throughput format and feasible in low-resource countries. For this purpose, HPV16/18 genotyping has previously been investigated as triage test in cervical cancer screening [7–9]. Additional molecular markers reflecting the underlying carcinogenic process are highly appealing alternatives. These include DNA copy number aberrations and DNA methylation changes in the host cell genome that result in altered coding and non-coding gene expression [3].

MicroRNAs (miRNAs) belong to an abundant class of small non-coding RNAs that post-transcriptionally regulate gene expression [10]. Altered expression of miRNAs has been shown to contribute to human malignancies, including cervical cancer, by influencing expression of oncogenes and tumor suppressor genes and subsequent deregulation of important intracellular pathways (reviewed by [11]). Given their short length of approximately 22 nucleotides, miRNAs are stable biomolecules and are less prone to degradation than their longer counterparts such as mRNAs or long non-coding RNAs [10, 12, 13]. It is therefore not surprising that miRNAs have been suggested and investigated as biomarkers for cancer diagnostics and prognostics [14–16].

To identify miRNAs that are relevant to cervical carcinogenesis, we previously performed an integrative screen combining array-based miRNA expression profiles with DNA copy number aberrations and DNA methylation [17, 18]. This resulted in the identification of five miRNAs for which differential expression was associated with frequently observed chromosomal gains of chromosomes 1q and 3q (miR-9-5p, miR-15b-5p, miR-28-5p) or losses of chromosome 11q (miR-100-5p, miR-125b-5p), and three miRNAs (miR-149-5p, miR-203a-3p, miR-375) for which gene silencing was mediated by DNA methylation of their respective promoter sequences (Table 1). Functional studies for miR-9, miR-203a, and miR-375 supported the biological relevance of these miRNA alterations, which were found to be involved in proliferation and anchorage independence of HPV-transformed cells [17–19].

**Table 1:** Candidate miRNAs

miRNA	Regulation [17]	Potential regulation mechanism [17, 18]	Class [17]
miR-9-5p	Up	Chromosomal gain (1q)	Late
miR-15b-5p	Up	Chromosomal gain (3q)	Late
miR-28-5p	Up	Chromosomal gain (3q)	Early continuous
miR-100-5p	Down	Chromosomal loss (11q)	Late
miR-125b-5p	Down	Chromosomal loss (11q)	Late
miR-149-5p	Down	DNA methylation	Early continuous
miR-203a-3p	Down	DNA methylation	Early continuous
miR-375	Down	DNA methylation	Late

In this study, we evaluated the clinical value of the eight either genetically or epigenetically deregulated miRNAs to serve as triage markers on cervical scrapes of hrHPV-positive women in cervical screening. For this purpose, we verified expression of the discovered 8 miRNAs in 58 cervical tissues and archival cervical scrapes of 225 hrHPV-positive women, built a predictive miRNA classifier, and evaluated its performance for the detection of high-grade CIN and cancer using leave-one-out cross-validation and ROC curve analysis.

## Methods

### Clinical specimens

Cervical tissue samples consisted of microdissected fresh frozen specimens, of which the majority has previously been used for miRNA microarray analysis [17]. In total, 8 normal cervical epithelial samples, 18 high-grade cervical intraepithelial neoplasia (CIN2–3) lesions, 22 cervical squamous cell carcinomas (SCC), and 11 adenocarcinomas (AC) were included. All but one normal sample were hrHPV-positive. The median age per group was as follows: normal, 35 years (range 31–47); CIN2–3, 34 years (range 26–54); SCC, 48.5 years (range 25–78); and AC, 39 years (range 31–64).

Cervical scrapes of 66 hrHPV-positive women without underlying disease (Pap 1) and 121 women with CIN3 were obtained from a screening population in the Utrecht region that had been collected between January 2010 and December 2011. Original 20 ml samples were concentrated and stored in 1 ml ThinPrep medium (Hologic, Vilvoorde, Belgium) at − 80 °C. For most samples, HPV genotyping was performed using the general primer GP5+/6 + −mediated PCR-enzyme immunoassay in combination with the luminex genotyping kit HPV GP at the time of sample collection [20, 21]. For samples with sufficient amounts of DNA for which no previous genotyping results were available, we used the HPV-Risk Assay (Self-screen BV, Amsterdam, The Netherlands) to complete our dataset [22]. Women without disease had a median age of 41 years (range 21–61). The median age of women with CIN3 was 35 years (range 22–60). HrHPV-positive scrapes from women with underlying cervical SCC (*n* = 29) and AC (*n* = 9, consisting of 7 AC and 2 adenosquamous carcinomas) [23, 24] were collected at the Antoni van Leeuwenhoek Hospital Amsterdam, The Netherlands, between January 2015 and March 2017. All cervical cancer samples were tested for hrHPV using the HPV-Risk Assay. Women with SCC had a median age of 51 years (range 29–86), and the median age of women with AC was 45 years (range 27–62).

### RNA isolation

Total RNA was isolated using TRIzol reagent (Thermo Fisher Scientific, Landsmeer, The Netherlands) according to the manufacturer’s instructions. The Qubit® microRNA Assay kit was used to quantify small RNA concentrations on a Qubit® 2.0 Fluorometer (both ThermoFisher Scientific).

### Quantitative RT-PCR

Expression of hsa-miR-9-5p, hsa-miR-15b-5p, hsa-miR-28-5p, hsa-miR-100-5p, hsa-miR-125b-5p, hsa-miR-149-5p, hsa-miR-203a-3p, and hsa-miR-375 was measured using TaqMan microRNA assays (000583, 000390, 000411, 000437, 000449, 002255, 000507, 000564; Thermo Fisher Scientific). For cervical scrapes, RNU24, RNU43, U6, U75, hsa-miR-423-3p, and hsa-miR-425-5p were included as potential reference genes (001001, 001095, 001973, 001219, 002626, 001516; Thermo Fisher Scientific).

Reverse transcription (RT) of all targets was multiplexed and validated in comparison to singleplex RT reactions (data not shown). In short, a primer pool was created by combining the specific RT primers. cDNA was synthesized from 20 ng small RNA template if available, for 5 samples the maximum possible amount (< 20 ng) of RNA was used. Each 16 μl reaction contained 6 μl primer pool, 0.3 μl dNTPs (100 mM), 1.5 μl RT buffer, 0.19 μl RNase inhibitor (20 U/μl), and 3 μl MultiScribe Reverse Trancriptase (TaqMan microRNA Reverse Transcription kit, Thermo Fisher Scientific). Quantitative PCR reactions were performed on the ViiA™ 7 Real-Time PCR System (Thermo Fisher Scientific) in a 384-well format. Each 10 μl reaction consisted of 5 μl TaqMan® Universal Master Mix II, 0.5 μl miRNA specific TaqMan assays (Thermo Fisher Scientific), 3.5 μl H_2_O, and 1 μl cDNA. Cycle conditions for cDNA synthesis and PCR were used according to the manufacturer’s protocols.

RNU24 and miR-423-3p were selected for normalization in cervical tissue samples and scrapes using our previously published strategy (data not shown) [25]. Data were normalized to the geometric mean Ct of both reference genes applying the ${2}^{-{\Delta \mathrm{C}}_{\mathrm{t}}}$ method [26]. All samples had a reference gene geometric mean Ct ≤ 32 and were therefore considered to be suitable for miRNA expression analysis.

### Statistical analysis

Statistical analysis was performed using R version 3.1.2. For logistic regression, R packages pROC and GRridge were used. The Spearman correlation coefficient (Rho) and associated *p* value was calculated to assess the agreement between qRT-PCR and previous microarray results. We performed an omnibus Kruskal-Wallis test to compare miRNA expression levels between normal, CIN3, SCC, and AC for each marker. Further, Wilcoxon rank test was applied with a significance level of 0.05 (two-sided) when the omnibus test showed a significant result (*p* < 0.05). *p* values from the post-hoc test were corrected with Benjamini-Hochberg correction method. Individual miRNA models and multi-miRNA classifiers for the detection of CIN3 were built performing univariable and multivariable logistic regression on square root transformed Ct ratios and evaluated using leave-one-out cross-validation. As a result, predicted probabilities, i.e., values between 0 and 1 representing the risk of an underlying CIN3, were calculated for each sample. For the construction of multi-miRNA classifiers, multivariable logistic regression analysis was followed by backward elimination to select relevant markers. Receiver-operated characteristic (ROC) curve analysis was carried out to evaluate the performance of the miRNA classifiers in detecting CIN3. For comparison of the obtained AUCs with a random classifier with AUC = 0.5, DeLong’s test for two correlated ROC curves was used [27].

### Cell culture, transfection, and cell viability assay of cervical cancer cell lines

Cervical cancer cell lines SiHa and CaSki were authenticated by STR testing using the Powerplex16 System (Promega, Leiden, The Netherlands) and cultured as described previously [28]. Cells were transiently transfected with 30 nM miRCURY LNA microRNA Power inhibitors for miR-15b-5p and negative control A (4103019, 199006; Exiqon, Vedbaek, Denmark) or 30 nM miRIDIAN microRNA mimics for miR-375 and negative control #2 (C-300682-05, CN-002000-01; GE Dharmacon, Lafayette, CO, USA). Cells were transfected with Dharmafect 1 (GE Dharmacon) for at least 6 h according to the manufacturer’s instructions. After transfection, cells were seeded in triplicate in 96-well plates (2500 cells/well). Cell viability was measured using the fluorometric CellTiter-Blue assay (Promega, Madison, WI, USA) according to the manufacturer’s protocol at days 0 and 2. The average measurement of day 0 was subtracted from the measurements at day 2. Each experiment was performed at least two times.

## Results

### Microarray-based differential miRNA expression in cervical tissue specimens can be verified by qRT-PCR

To confirm our previously obtained microarray results, we used qRT-PCR to determine the expression levels of the eight genetically or epigenetically deregulated miRNAs in normal cervical squamous epithelium (*n* = 8), high-grade CIN lesions (CIN2–3, *n* = 18), SCC (*n* = 22), and AC (*n* = 11) tissue specimens, of which 44 had also been analyzed by microarray [17]. Except for miR-28-5p and miR-100-5p (Spearman correlation coefficient (Rho) = 0.521 and Rho = 0.645, respectively), qRT-PCR results strongly correlated with microarray results as indicated by Rho > 0.75 (Additional file 1: Figure S1). Because of the low correlations observed for miR-28-5p and miR-100-5p, both miRNAs were excluded from further analysis. Significantly differential expression between normal and CIN2-3 could be verified for 1 out of 2 upregulated miRNAs (miR-9-5p) and for 2 out of 4 downregulated miRNAs (miR-149-5p, miR-203a-3p; Additional file 2: Figure S2 and Additional file 3: Table S1). Downregulation of miR-375 in CIN2-3 compared to normal was borderline significant (*p* = 0.067). All miRNAs showed significantly differential expression between normal and SCC.

### Differential miRNA expression is reflected in cervical scrapes

**Fig. 1:**
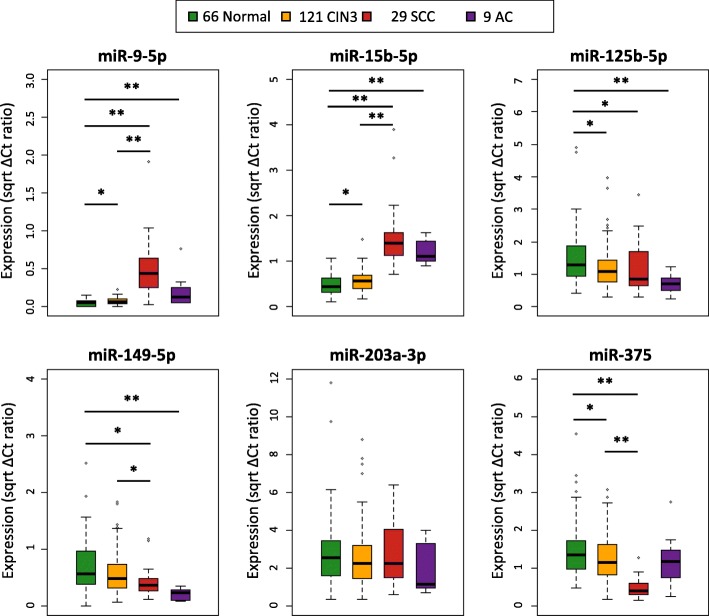
Differential expression of selected miRNAs in cervical scrapes. qRT-PCR results were normalized to RNU24 and miR-423, and all values were square root transformed. *p < 0.05, **p < 0.005

Next we analyzed whether altered expression of candidate miRNAs is also detectable in cervical scrapes. HrHPV-positive scrapes of women without cervical disease (normal, *n* = 66) or with underlying CIN3 (*n* = 121), SCC (*n* = 29), or AC (*n* = 9) were analyzed by qRT-PCR. All six miRNAs except for miR-9-5p could be detected in at least 99% of samples. MiR-9-5p remained undetected in about one fourth (23%, 49/212) of samples. Low expression levels of miR-9-5p were consistent with microarray and qRT-PCR results obtained in cervical tissue specimens (Fig. 1, Additional file 2: Figure S2). Because reproducibility is reduced and technical PCR noise increases when amplifying lowly abundant transcripts [29–31], we did not consider miR-9-5p a suitable biomarker and excluded it from further analysis. MiRNA expression results obtained in hrHPV-positive cervical scrapes were generally comparable to those obtained in tissue samples (Fig. 1, Additional file 2: Figure S2 and Additional file 4: Table S2). Expression of miR-15b-5p was significantly increased in scrapes of women with CIN3 compared to controls, while miR-125b-5p and miR-375 were significantly downregulated in scrapes of women with CIN3. No significant difference between normal and CIN3 was observed for miR-149-5p and miR-203a-3p. Similar to observations in tissue samples, the largest expression change between scrapes of women with CIN3 and SCC was observed for upregulated miR-15b-5p. Expression of miR-149-5p and miR-375 was significantly decreased in scrapes of women with SCC compared to normal and CIN3. Comparison of normal controls to AC showed a significant increase in expression of all upregulated miRNAs in scrapes of AC patients. In accordance with tissue results, miR-125b-5p and miR-149-5p were significantly downregulated between normal and AC, and miR-375 did not show a significant difference between normal scrapes and those of women with AC.

### Predictive miRNA classifier detects large subset of CIN3 lesions

**Fig. 2:**
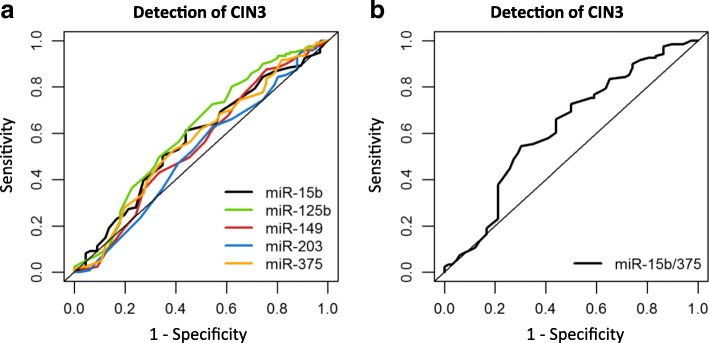
ROC curve analysis of miRNA classifiers for the detection of CIN3. Results obtained from 66 hrHPV-positive scrapes from women without underlying disease and 121 scrapes from women with CIN3 were used to build (**a**) individual miRNA classifiers and (**b**) a 2-miRNA classifier. Classifiers were validated by leave-one-out cross-validation

Univariate logistic regression analysis was carried out on expression results of the five remaining markers (miR-15b-5p, miR-125b-5p, miR-149-5p, miR-203a-3p and miR-375) obtained from hrHPV-positive cervical scrapes of women without cervical disease and women with underlying CIN3, and validated by leave-one-out cross-validation. Single miRNAs achieved areas under the curve (AUC) varying from 0.523 (miR-203a-3p) to 0.605 (miR-125b-5p, Table 2, Fig. 2a).

**Table 2:** Comparison of optimal sensitivity and specificity between miRNA panels for the detection of CIN3 based on leave-one-out cross-validation

Panel	AUC	Cutoff	Sensitivity (%)	Specificity (%)	*p* value^*^
Single markers					
miR-15b-5p	0.573	0.629	62.0	56.1	0.098
miR-125b-5p	0.605	0.641	72.7	47.0	0.020
miR-149-5p	0.542	0.597	84.3	28.8	0.356
miR-203a-3p	0.523	0.654	62.0	48.5	0.619
miR-375	0.565	0.671	52.9	62.1	0.145
Two markers					
miR-15b-5p/375	0.622	0.682	54.5	69.7	0.006

^*^p value: comparison between the miRNA classifier and a random classifier with an AUC of 0.5

*CIN* cervical intraepithelial neoplasia, *AUC* area under the curve

To determine the most discriminative miRNA marker panel for CIN3, we performed multivariable logistic regression analysis followed by backward marker selection and validated results by leave-one-out cross-validation. This resulted in a 2-miRNA classifier consisting of miR-15b-5p and miR-375 with an AUC of 0.622 and optimal sensitivity and specificity of 55 and 70%, respectively (Table 2, Fig. 2b). Adding additional miRNAs to the 2-marker panel did not further improve performance.

### MiRNA classifier detects all cervical carcinomas

To test how our miRNA classifiers performed in the detection of cervical carcinomas, we applied the previously determined regression models and corresponding cutoffs to our results obtained on cervical scrapes of women with underlying SCC or AC. The 2-miRNA classifier detected all SCC and all AC (Table 3). Overall, the 2-miRNA classifier achieved a CIN3+ detection rate of 65% at 70% specificity.

**Table 3:** Sensitivity of miRNA panels for the detection of SCC and AC

Panel	Sensitivity %	
	Detection of SCC	Detection of AC
Single markers		
miR-15b-5p	100	100
miR-125b-5p	69.0	100
miR-149-5p	93.1	100
miR-203a-3p	55.2	66.7
miR-375	96.6	55.6
Two markers		
miR-15b-5p/375	100	100

*SCC* squamous cell carcinoma, *AC* adenocarcinoma

### HPV16/18 genotyping in conjunction with our miRNA classifier improves CIN3 detection

**Fig. 3:**
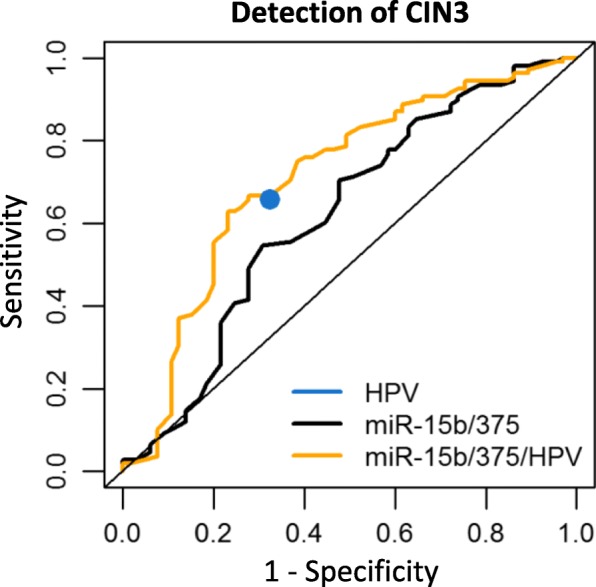
ROC curve analysis of HPV16/18 genotyping and the 2-miRNA classifier for the detection of CIN3. Results obtained from scrapes with known HPV16/18 genotyping results (65 normal, 108 CIN3) were used to build classifiers for HPV16/18 genotyping (HPV), a new 2-miRNA classifier (miR-15b/375) and the 2-miRNA classifier combined with HPV16/18 genotyping (miR-15b/375/HPV). Classifiers were validated by leave-one-out cross-validation. The model miR-15b/375/HPV is significantly better than the 2-miRNA classifier (*p* = 0.011)

To compare the performance of our 2-miRNA classifier to HPV16/18 genotyping, samples were either classified as HPV16/18 positive (19 normal, 71 CIN3) or other hrHPV type positive (46 normal, 37 CIN3), for those samples for which the genotype was known (*n* = 173 out of 187). For this smaller sample set, we built (i) a new 2-miRNA classifier consisting of miR-15b and miR-375 and (ii) a logistic regression model combining the 2-miRNA classifier with HPV16/18 genotyping. Consistent with previous reports, HPV16/18 genotyping achieved 66% sensitivity and 68% specificity for CIN3 detection (Table 4) [7, 32]. The 2-miRNA classifier obtained in the smaller sample set had a comparable performance to the one obtained from the entire set of samples (Tables 3 and 4). While HPV16/18 genotyping alone was inferior to the 2-miRNA classifier (*p* = 5.2 e-16, Fig. 3), a classifier combining our two selected miRNAs with HPV16/18 genotyping had an improved performance and achieved an AUC of 0.712 (Table 4, Fig. 3). The 2-miRNA classifier adjusted by HPV16/18 type had a significantly better performance than the 2-miRNA classifier (*p* = 0.011). Including HPV16/18 genotyping in the classifier increased both sensitivity and specificity to 63 and 77%, respectively, and all SCC and AC were detected (data not shown).

**Table 4:** Optimal sensitivity and specificity for the detection of CIN3 for hrHPV type (HPV16/18, others) and the miRNA classifier in conjunction with hrHPV type based on a smaller sample set with known hrHPV type infection and leave-one-out cross-validation

Panel	AUC	Cutoff	Sensitivity %	Specificity %	*p* value^*^
Single marker					
HPV type	0.445	n.a.	65.7	67.7	0.266
Multiple markers					
miR-15b-5p/375	0.622	0.656	55.6	69.2	0.008
miR-15b-5p/375/HPV	0.712	0.666	63.0	76.9	5.8 e-07

^*^p value: comparison between the miRNA classifier and a random classifier with an AUC of 0.5

*CIN* cervical intraepithelial neoplasia, *AUC* area under the curve, *n.a* not applicable

### Knockdown of miR-15b-5p and ectopic expression of miR-375 reduces viability in cervical cancer cells

**Fig. 4:**
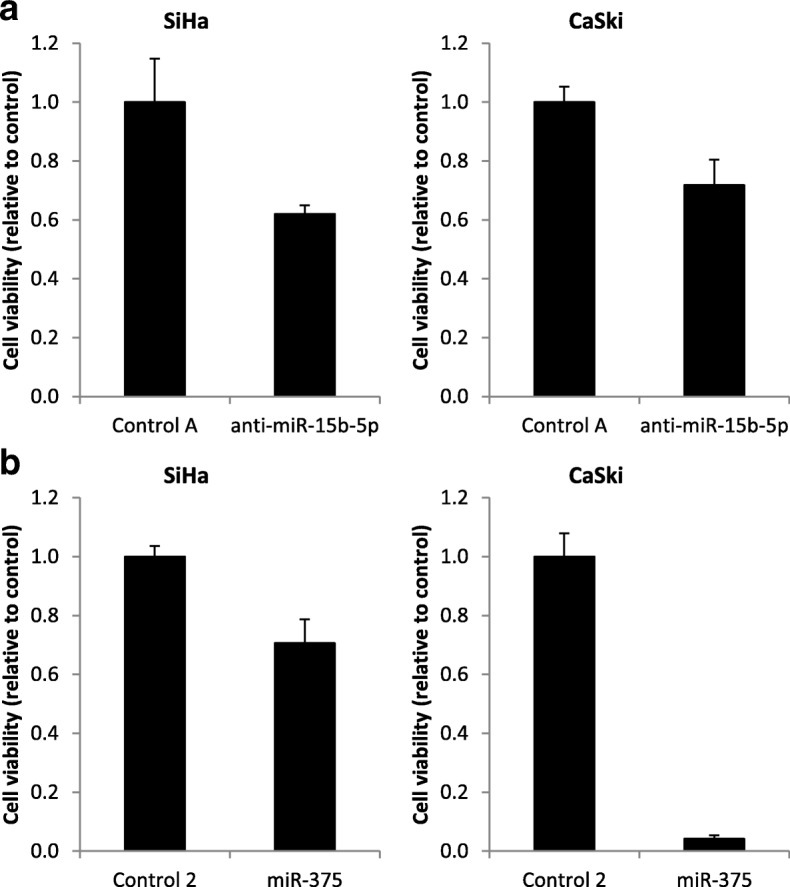
Functional effect of our selected marker miRNAs in cervical cancer cell lines. Cell viability of SiHa and CaSki cells upon (**a**) knockdown of miR-15b-5p and (**b**) ectopic expression of miR-375. Results are representative of two independent experiments

Next, we investigated whether our miRNA markers are directly involved in cervical carcinogenesis, as is suggested by their genetic (miR-15b-5p) or epigenetic (miR-375) regulation. Transfection of cervical cancer cell lines SiHa and CaSki with miR-15b-5p inhibitors led to a reduction in cell viability, suggesting that miR-15b-5p acts as an oncomiR (Fig. 4a). Ectopic expression of miR-375 in SiHa and CaSki cells significantly reduced cell viability, supporting its role as tumor suppressive miRNA (Fig. 4b).

## Discussion

In this study, we analyzed the triage capacity on hrHPV-positive cervical scrapes of a panel of miRNAs that exhibit either genetically or epigenetically mediated expression changes in cervical precancerous and cancerous tissue specimens and that in part have also been shown to be functionally involved in cervical carcinogenesis [17, 18]. We found that expression patterns detected in cervical tissue samples were reflected in cervical scrapes. By logistic regression analysis, a 2-miRNA classifier was built that at 70% specificity achieved 55% sensitivity for the detection of CIN3 and 100% sensitivity for the detection of SCC and AC. Upon inclusion of HPV16/18 genotyping, the sensitivity and specificity for CIN3 detection could be increased to 63 and 77%, respectively. Our data suggest that miRNA expression analysis offers a promising alternative molecular tool to triage hrHPV-positive women.

In a systematic review, Sharma et al. identified a total of 246 miRNAs that become deregulated in cervical cancer, with miR-21, miR-143, miR-145, miR-203, miR-214, and miR-218 being the most frequently described [33]. Most published studies identified deregulated miRNAs in cervical tissue samples by microarray analysis [17, 34, 35]. Studies focusing on the diagnostic or prognostic use of miRNAs detected by qRT-PCR in cervical biopsies are numerous, as are studies investigating the functional contribution of individual miRNAs to cervical cancer development. Data on the clinical applicability of miRNA expression analysis in cervical scrapes for cervical screening purposes, however, is limited. To the best of our knowledge, only Tian et al. published on the analysis of a panel of candidate miRNA markers in HPV-positive scrapes of a gynecology outpatient clinic population to date [36]. Candidate miRNAs analyzed by Tian et al. have previously been shown to become differentially expressed during disease progression. A combination of miR-375 and miR-424 resulted in an AUC value of 0.853 for the detection of CIN3+, showing the promise of biologically relevant miRNAs as disease markers. Importantly, our study as well as the study by Tian et al. shows that the use of more than one miRNA improves detection of cervical disease. Although miR-15b-5p alone detected all carcinomas, combining miR-15b-5p with miR-375 proved beneficial for the detection of CIN3 without a loss of sensitivity for SCC and AC. The fact that the individual miRNA performing best in the detection of CIN3 (miR-125b-5p) was not included in the 2-miRNA classifier further demonstrates that analysis of multiple complementary miRNAs can improve the detection of cervical disease. Importantly, we here show that combining miRNA profiling with HPV16/18 genotyping can further improve the detection of cervical precancer. This is especially attractive, as HPV16/18 genotyping is frequently included in clinically validated HPV tests [20, 37] and HPV16/18 genotyping and miRNA expression analysis can be performed on the same cervical scrape.

In line with Tian et al., our study shows that downregulated miRNAs can be suitable biomarkers, although the selection of downregulated markers may seem counterintuitive at first. It is important to note that the relative decrease in expression observed with cervical disease progression does not give an indication of the absolute abundance of a miRNA. Differences in performance between the study of Tian et al. and ours are most likely due to differences between study populations. While our cohort of cervical scrapes was obtained from a screening population, Tian et al. analyzed scrapes from a clinic-based referral population, which potentially contains more advanced CIN lesions. Altered expression of our candidate miRNAs is caused by either genetic or epigenetic changes which have previously been shown to be associated with cervical cancer and so-called advanced CIN3 lesions [3]. Their association with progression risk to cancer could explain why our miRNA classifiers detect only a subset of CIN3 lesions. Our 2-miRNA classifier detected all cervical cancers and 55% of CIN3 at 70% specificity, a generally accepted specificity for triage markers [38]. At present, adopted triage options for HPV-positive women include reflex cytology, HPV16/18 genotyping, repeat HPV testing, and/or repeat cytology. While triage by the current miRNA panel does not yet meet the criteria for acceptability of a triage strategy [39], we here show that triage by miRNA expression analysis is feasible and offers a promising alternative. MiRNA analysis is objective, highly reproducible, and can be performed in a high-throughput manner. We do acknowledge that further panel optimization is required and expect that analysis of additional miRNAs will result in a miRNA classifier with improved performance. How an optimized miRNA panel performs in comparison to or as adjunct to cytology, HPV16/18 genotyping, DNA methylation markers, and/or other cellular markers such as p16^INK4A^/Ki-67 will be subject of future studies.

Overexpression of miR-15b-5p has previously been associated with HPV-induced malignancies including cervical cancer, tonsillar tumors, and anal carcinomas [40, 41]. Moreover, miR-15b-5p was shown to promote cell viability, migration, and invasion in non-small cell lung cancer by targeting metastasis suppressor TIMP2 [42]. In line with this, we observed reduced cervical cancer cell viability upon knockdown of miR-15b-5p suggestive of an oncogenic role for this miRNA. Expression of miR-375 in cervical cancer, on the other hand, is downregulated by increased DNA methylation as well as by a frequently occurring focal loss of chromosome 2q35 [18, 19, 43, 44]. In line with literature, we found that ectopic expression of miR-375 in hrHPV-positive cervical cancer cell lines reduces cell viability [19, 45]. Jung et al. previously showed that miR-375 restored major tumor suppressors p53, p21, and RB levels by deregulation of HPV16 and HPV18 viral transcripts and directly targeting E6AP [45].

One limitation of this study is that we did not include HPV-positive scrapes of women diagnosed with CIN1 and CIN2. Further validation of our 2-miRNA classifier is needed in an independent population-based screening cohort consisting of consecutive hrHPV-positive cervical scrapes including CIN1 and CIN2 lesions. In addition, the samples used in our study had been stored at room temperature for at least 1 year and at − 80 °C for another 3 to 4 years, and clinical material was limited. We showed that using as little as 20 ng of small RNA still enables the early detection of cervical cancer, but we cannot exclude that higher amounts of RNA, as also used by Tian et al., may give a better discrimination between normal and CIN3. On the other hand, our data also demonstrate that miRNAs are very stable molecules. This is of particular importance in screening settings where cervical scrape material is send to central diagnostic laboratories for molecular testing. While we analyzed a selected panel of eight miRNAs which were shown to become genetically or epigenetically deregulated during cervical carcinogenesis in cervical tissue samples, candidate miRNAs should ideally be selected directly from whole miRNome data obtained from cervical scrapes [46]. Future studies will therefore aim to identify an optimal panel of miRNAs for the detection of CIN3 and cancer.

## Conclusions

In conclusion, present data show that analysis of differentially expressed miRNAs may provide an alternative molecular triage strategy in hrHPV-based cervical screening. Our data indicate that miRNAs that are genetically or epigenetically deregulated during and directly involved in disease progression are promising biomarkers for the detection of cervical cancer and a subset of CIN3 lesions. CIN3 detection was further improved by inclusion of HPV16/18 genotyping. Further optimization of the marker panel and validation in an independent cohort of hrHPV-positive cervical scrapes will reveal whether triage of hrHPV-positive women by miRNA expression analysis offers an objective and economical alternative to cytology.

## Additional files

Additional file 1: **Figure S1.** Correlation between microarray and qRT-PCR results for the eight selected miRNAs [17]. Results are shown for cervical tissue specimens of women without disease (normal, *n* = 9), with precancer (CIN2–3, *n* = 18), squamous cell carcinoma (SCC, *n* = 9), and adenocarcinoma (AC, *n* = 9). Linear regression is indicated by the black line and Spearman correlation coefficients (Rho) are shown. qRT-PCR results were normalized to RNU24 and miR-423 and all values were log2 transformed. (PDF 270 kb)

Additional file 2: **Figure S2.** Differential expression of selected miRNAs in cervical tissue specimens. qRT-PCR results were normalized to RNU24 and miR-423, and all values were square root transformed. *p < 0.05, **p < 0.005. (PDF 179 kb)

Additional file 3: **Table S1.** *p* values of differentially expressed miRNAs in tissue samples. *p* values were determined by Wilcoxon rank test and corrected applying the Benjamini-Hochberg correction method for multiple testing. qRT-PCR results obtained from normal squamous epithelium (*n* = 8), CIN2–3 (*n* = 18), SCC (*n* = 22), and AC (*n* = 11) were included in the analysis. (PDF 262 kb)

Additional file 4: **Table S2.** *p* values of differentially expressed miRNAs in cervical scrapes. *p* values were determined by Wilcoxon rank test and were corrected by applying the Benjamini-Hochberg correction method for multiple testing. qRT-PCR results obtained from cervical scrapes of women without disease (*n* = 66), CIN2–3 (*n* = 121), SCC (*n* = 29), or AC (*n* = 9) were included in the analysis. (PDF 262 kb)

## Abbreviations

(hr)HPV: (High-risk) Human papillomavirus
AC: Adenocarcinoma
AUC: Area under the curve
CIN: Cervical intraepithelial neoplasia
E6AP: E6-associated protein
miRNA: MicroRNA
p21: Cyclin dependent kinase inhibitor 1A
p53: Tumor protein p53
RB: Retinoblastoma protein
ROC: Receiver-operated characteristic
SCC: Squamous cell carcinoma
