# Supplementary figures and images for: Triage of high-risk HPV-positive women in population-based screening by miRNA expression analysis in cervical scrapes; a feasibility study
Source: Clin Epigenetics. 2018 Jun 7;10:76. doi: 10.1186/s13148-018-0509-9 (PMC5992707; doi:10.1186/s13148-018-0509-9)

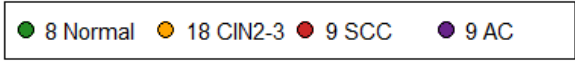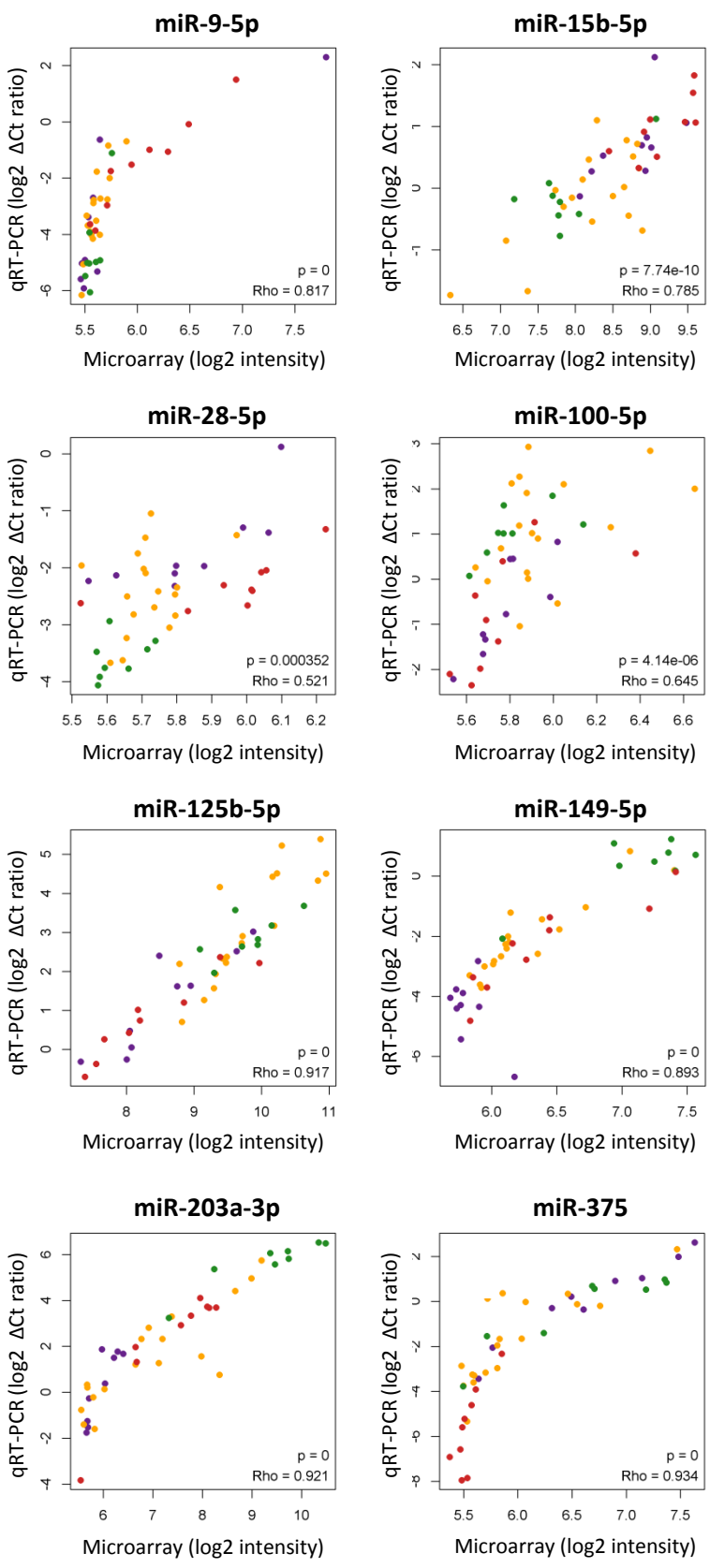

Supplement: Supplementary file 1 — Figure S1. Correlation between microarray and qRT-PCR results for the eight selected miRNAs [17]. Results are shown for cervical tissue specimens of women without disease (normal, n = 9), with precancer (CIN2–3, n = 18), squamous cell carcinoma (SCC, n = 9), and adenocarcinoma (AC, n = 9). Linear regression is indicated by the black line and Spearman correlation coefficients (Rho) are shown. qRT-PCR results were normalized to RNU24 and miR-423 and all values were log2 transformed. (PDF 270 kb) [file 13148_2018_509_MOESM1_ESM.pdf]

8 Normal 18 CIN2-3 22 SCC 11 AC

miR-9-5p

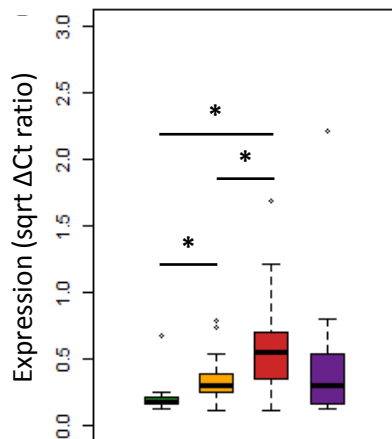

miR-15b-5p

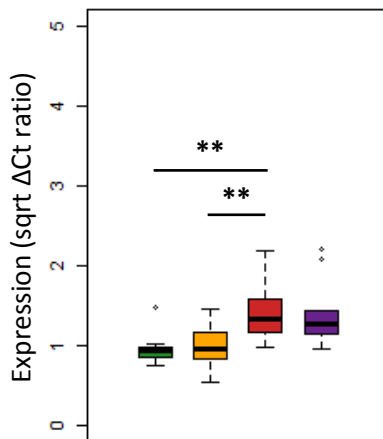

miR-125b-5p

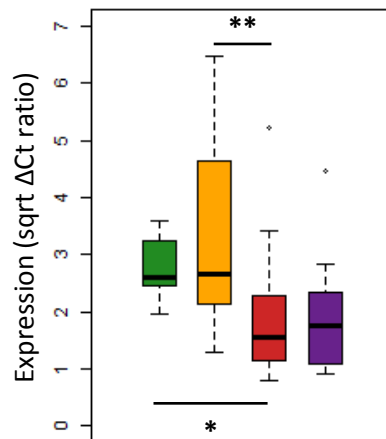

miR-149-5p

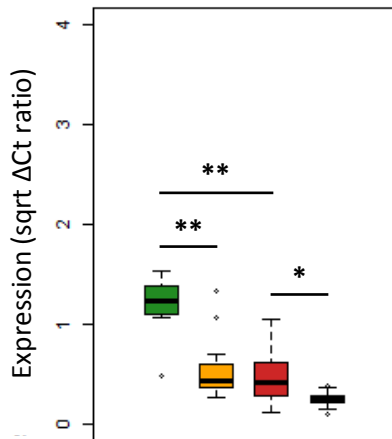

miR-203a-3p

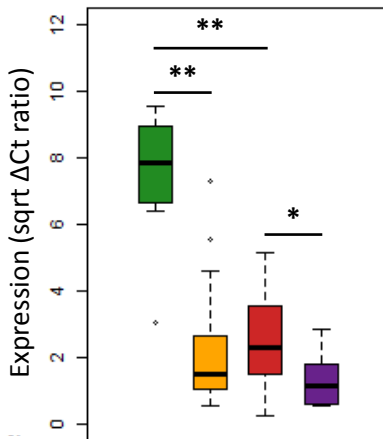

miR-375

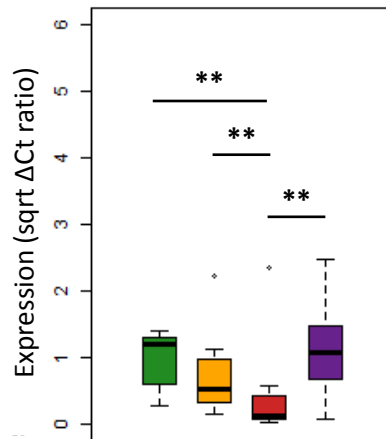

Supplement: Supplementary file 2 — Figure S2. Differential expression of selected miRNAs in cervical tissue specimens. qRT-PCR results were normalized to RNU24 and miR-423, and all values were square root transformed. *p < 0.05, **p < 0.005. (PDF 179 kb) [file 13148_2018_509_MOESM2_ESM.pdf]
