# Supplementary material for: Triage of high-risk HPV-positive women in population-based screening by miRNA expression analysis in cervical scrapes; a feasibility study
Source: Clin Epigenetics. 2018 Jun 7;10:76. doi: 10.1186/s13148-018-0509-9 (PMC5992707; doi:10.1186/s13148-018-0509-9)
Supplement: Supplementary file 3 — Table S1. p values of differentially expressed miRNAs in tissue samples. p values were determined by Wilcoxon rank test and corrected applying the Benjamini-Hochberg correction method for multiple testing. qRT-PCR results obtained from normal squamous epithelium (n = 8), CIN2–3 (n = 18), SCC (n = 22), and AC (n = 11) were included in the analysis. (PDF 262 kb) [file 13148_2018_509_MOESM3_ESM.pdf]

Additional file 2: Table S1. P-values of differentially expressed miRNAs in cervical tissue samples. P-values were determined by Wilcoxon rank test and corrected applying the Benjamini-Hochberg correction method for multiple testing. qRT-PCR results obtained from normal squamous epithelium (n=8), CIN2-3 (n=18), SCC (n=22) and AC (n=11) were included in the analysis.

miRNA	*Kruskal Wallis*	*CIN2-3 vs normal*	*SCC vs normal*	*SCC vs CIN2-3*	*SCC vs AC*
Upregulated					
miR-9-5p	**0.009**	**0.034**	**0.007**	**0.034**	0.170
miR-15b-5p	**0.000**	0.849	**0.000**	**0.000**	0.849
Downregulated					
miR-125b-5p	**0.001**	0.935	**0.007**	**0.003**	0.849
miR-149-5p	**0.000**	**0.000**	**0.000**	0.537	**0.007**
miR-203a-3p	**0.000**	**0.000**	**0.000**	0.232	**0.016**
miR-375	**0.000**	0.067	**0.000**	**0.000**	**0.000**

CIN, cervical intraepithelial neoplasia; SCC, squamous cell carcinoma; AC, adenocarcinoma; p-values < 0.05 in bold.
