# Supplementary material for: Triage of high-risk HPV-positive women in population-based screening by miRNA expression analysis in cervical scrapes; a feasibility study
Source: Clin Epigenetics. 2018 Jun 7;10:76. doi: 10.1186/s13148-018-0509-9 (PMC5992707; doi:10.1186/s13148-018-0509-9)
Supplement: Supplementary file 4 — Table S2. p values of differentially expressed miRNAs in cervical scrapes. p values were determined by Wilcoxon rank test and were corrected by applying the Benjamini-Hochberg correction method for multiple testing. qRT-PCR results obtained from cervical scrapes of women without disease (n = 66), CIN2–3 (n = 121), SCC (n = 29), or AC (n = 9) were included in the analysis. (PDF 262 kb) [file 13148_2018_509_MOESM4_ESM.pdf]

Additional file 3: Table S2. P-values of differentially expressed miRNAs in cervical scrapes. P-values were determined by Wilcoxon rank test and corrected applying the Benjamini-Hochberg correction method for multiple testing. qRT-PCR results obtained from cervical scrapes of women without disease (n=66), CIN2-3 (n=121), SCC (n=29) or AC (n=9) were included in the analysis.

miRNA	*Kruskal Wallis*	*CIN3 vs normal*	*SCC vs normal*	*SCC vs CIN3*	*AC vs normal*
Upregulated					
miR-9-5p	**0.000**	**0.044**	**0.000**	**0.000**	**0.002**
miR-15b-5p	**0.000**	**0.037**	**0.000**	**0.000**	**0.000**
Downregulated					
miR-125b-5p	**0.001**	**0.006**	**0.044**	0.586	**0.002**
miR-149-5p	**0.000**	0.095	**0.005**	**0.037**	**0.000**
miR-203a-3p	0.155	0.158	0.677	0.528	0.095
miR-375	**0.000**	**0.044**	**0.000**	**0.000**	0.294

CIN, cervical intraepithelial neoplasia; SCC, squamous cell carcinoma; AC, adenocarcinoma; p-values < 0.05 in bold.
